# Theory and Practice of Obstetrics

**Published:** 1886-05

**Authors:** 


Theory and Practice of Obstetrics. *By* P. Careoux *and* S. Tarnier. *Edited and revised by* Robert J. Hess, m. d., *Philadelphia. Octavo, pp. xxxii., 1081. Philadelphia:* P. Blakiston Son & Co. 1885. *Chicago:* W. T. Keener.

Cazeaux's "Obstetrics"—a justly popular text-book in the United States some years ago—possesses now the historical interest to which as a classical work it is entitled. The additions and revisions of Professor Tarnier fail to bring the book up to the standard established by recent publications on the same subject in the English, German and French languages.

A similar remark is applicable to the editorial labors of Dr. Hess, although the value of the work is enhanced by his very readable chapter on puerperal insanity. The chapter on laceration of the perineum is entirely superfluous.

Due credit is nowhere given to Schroeder, Braune, Bandl and others for the free use of their plates and figures.

The book is essentially an old one. The recent anatomical investigations of the *cervix uteri*, during pregnancy and labour,

Professor Lahs' theory of labour, the results of late researches into the nature of eclampsia and puerperal fever, and the like, are conspicuous by their absence.

